# The economic burden experienced by carers of children who had a critical deterioration at a tertiary children’s hospital in the United Kingdom (the DETECT study): an online survey

**DOI:** 10.1186/s12887-023-04268-8

**Published:** 2023-08-31

**Authors:** Eduardo Costa, Céu Mateus, Bernie Carter, Sarah Siner, Dawn Jones, Leah Evans, Jenny Preston, Fulya Mehta, Caroline Lambert, Bruce Hollingsworth, Enitan D. Carrol, Gerri Sefton

**Affiliations:** 1 Nova School of Business and Economics, Carcavelos, Portugal; 2 Lancaster University, Lancaster, UK; 3 Faculty of Health, Social Care and Medicine, Edge Hill University, Ormskirk, UK; 4 Clinical Research Division, Alder Hey Children’s NHS Foundation Trust, Liverpool, UK; 5 University of Liverpool, Liverpool, UK; 6 Alder Hey Children’s NHS Foundation Trust, Liverpool, UK; 7 Institute of Infection, Veterinary and Ecological Sciences, University of Liverpool, Liverpool, UK; 8 Dept of Infectious Diseases, Alder Hey Children’s NHS Foundation Trust, Liverpool, UK; 9 Intensive Care Unit, Alder Hey Children’s NHS Foundation Trust, Liverpool, UK

**Keywords:** Economic burden, Productivity loss, Critical deterioration events, Paediatric children critical care

## Abstract

**Background:**

Unplanned critical care admissions following in-hospital deterioration in children are expected to impose a significant burden for carers across a number of dimensions. One dimension relates to the financial and economic impact associated with the admission, from both direct out-of-pocket expenditures, as well as indirect costs, reflecting productivity losses. A robust assessment of these costs is key to understand the wider impact of interventions aiming to reduce in-patient deterioration. This work aims to determine the economic burden imposed on carers caring for hospitalised children that experience critical deterioration events.

**Methods:**

Descriptive study with quantitative approach. Carers responded to an online survey between July 2020 and April 2021. The survey was developed by the research team and piloted before use. The sample comprised 71 carers of children admitted to a critical care unit following in-patient deterioration, at a tertiary children’s hospital in the UK. The survey provides a characterisation of the carer’s household and estimates of direct non-medical costs grouped in five different expenditure categories. Productivity losses can also be estimated based on the reported information.

**Results:**

Most carers reported expenditures associated to the child’s admission in the week preceding the survey completion. Two-thirds of working carers had missed at least one workday in the week prior to the survey completion. Moreover, eight in ten carers reported having had to travel from home to the hospital at least once a week. These expenditures, on average, amount to £164 per week, grouped in five categories (38% each to travelling costs and to food and drink costs, with accommodation, childcare, and parking representing 12%, 7% and 5%, respectively). Additionally, weekly productivity losses for working carers are estimated at £195.

**Conclusion:**

Unplanned critical care admissions for children impose a substantial financial burden for carers. Moreover, productivity losses imply a subsequent cost to society. Even though subsidised hospital parking and on-site accommodation at the hospital contribute to minimising such expenditure, the overall impact for carers remains high. Interventions aiming at reducing emergency critical care admissions, or their length, can be crucial to further contribute to the reduction of this burden.

**Trial Registration:**

Current Controlled Trials ISRCTN61279068, date of registration 07/06/2019, retrospectively registered.

**Supplementary Information:**

The online version contains supplementary material available at 10.1186/s12887-023-04268-8.

## Background

The hospitalisation of a child creates an emotional burden for carers [1]. Clinical deterioration requiring emergency escalation of care is likely to increase that psychological burden substantially. These effects are likely to be more profound for children and parents whose transfer to high dependency or paediatric intensive care units was unplanned, compared to planned transfers, e.g. following major elective surgery. Surrounded by uncertainty, carers of these children can experience anxiety and depression [2–4].

Despite the emotional burden faced by carers, typically, their regular presence and caregiving skills at their child’s bedside are well received by health professionals [5]. Their presence in the hospital setting can contribute to enhancing the recovery of their children [6].

To be able to spend time with their child in the hospital, carers need to modify their lifestyle [7, 8]. This has implications regarding their working status and their daily routine, particularly for carers of children with a long-term condition [9, 10]. For instance, a study in the UK suggests that over one third of mothers gave up all paid work following their child’s cancer diagnosis [11]. Even in the short term, a child’s hospitalisation can financially impact carers, and also imposes a productivity loss for society [12].

Moreover, changes to the daily routine generated by the child’s hospitalisation are likely to expose carers to additional out-of-pocket direct expenditures [13]. Even in a publicly financed healthcare system, such as the UK NHS, users are faced with expenses due to transportation, accommodation, food, among others. These costs are magnified when a child is admitted to a specialised paediatric facility situated far from their home. A recent study in Ireland suggests that travel, parking, accommodation, food and childcare costs are among the main expenditures paid directly by families - direct out-of-pocket payments [14]. Literature suggests considerable time and financial resources expended by families caring for hospitalised children [15]. For children with cancer, the literature suggests also high economic burden faced by carers [16]. Moreover, this burden increases with treatment complexity and among families with younger children [12]. The literature on the economic burden for children admitted in critical care units is scarcer. Still, existing estimates suggest considerable out-of-pocket expenditures faced by carers [8] and additional work productivity impact [17].

Overall, emergency critical care admissions following in-hospital deterioration in children are expected to increase the hospital costs for care delivered. These events also impose a significant burden for carers, which is poorly acknowledged in the literature.

The Dynamic Electronic Tracking and Escalation to reduce critical Care Transfers (DETECT) study [18], implemented a proactive SMART technology end-to-end deterioration solution (the DETECT surveillance system) across a tertiary children’s hospital. The DETECT surveillance system aims to proactively screen paediatric patients for early signs of serious deterioration or sepsis, thereby reducing complications and emergency transfers to critical care following deterioration in hospital. Part of the analysis plan is to evaluate the cost-effectiveness of the technology at reducing critical deterioration. During the Patient and Public involvement (PPI) process contributing to this research proposal, parents were very vocal that the cost-effectiveness component of the study should report wider than the direct hospital costs associated with in-patient deterioration and should also incorporate the impact on families. Therefore, the aim of this paper is to provide an assessment of the economic burden imposed on carers of hospitalised children that experience critical deterioration events, specifically the direct out-of-pocket expenditures, as well as indirect productivity losses for carers of children admitted to critical care units.

## Methods

### Design and administration of the survey

The health economic evaluation of the DETECT technology used in the study balanced the cost of the technology against the cost and productivity gains for the hospital. However, a relevant dimension relates to measuring the cost to carers whose child has a critical deterioration. In this study, these carers are primarily the children’s parents.

An online survey^1^ was conducted with the goal of assessing the carers’ personal situation. This was an essential tool to estimate both the expenditures and the productivity losses incurred by carers due to children’s hospitalisations. The survey included 18 questions to provide a characterisation of the carer household and costs. Different indicators were also collected to estimate the burden associated with these hospitalisations and productivity losses. Identifiers were used to link to the hospital case number for each critical deterioration. This allowed for demographic data including postcode to be recorded. Details on the survey questions can be found in appendix 1.

Ethics approval was given for the study, by the regional ethics committee, REC 17/NW/0533, and the Health Research Authority permission to proceed was issued, IRAS ID 215,339. After agreement on the final version was reached, a pre-final final version was piloted with carers advisors. Following feedback received, amendments were made, and it was released. The survey was developed on Qualtrics platform, allowing surveys to be completed on computers or mobile devices.

Dedicated DETECT study research nurses underwent training before the dissemination of the survey. Recruitment occurred between July 2020 and April 2021 at Alder Hey Children’s Hospital, a tertiary setting in Liverpool in the UK. Carers of children who experienced critical deterioration events during their hospitalisation were invited by the DETECT research nurses to participate in the survey. Carers were provided information about the purposes of the survey and were asked to sign a consent form. The data were uploaded to the secure Qualtrics platform at Lancaster University. The survey data were downloaded from the Qualtrics platform and analysed using Excel and Stata 13.1.

### Estimating direct out-of-pocket expenditures

Out-of-pocket expenditures are costs paid directly by the carers, incurred due to their children’s hospitalisation. Costs were assessed for five different categories and stated in British Pounds (£). Individuals were asked to report average costs for five different categories (travelling, parking, accommodation, food & drink, and childcare). Carers were asked to report costs experienced during the week preceding the survey completion. Overall, there was very few missing data. Adjustments and inference were required to provide a more accurate estimate on the costs faced by carers. Since costs were reported for two different years (2020 and 2021), they were adjusted for inflation to allow for a proper comparison. Moreover, total costs were also reported as a fraction of total estimated weekly wage, to provide an indication of the degree of financial strain faced by each carer.

Travelling costs were either reported directly by the carer or estimated based on the distance reported between home and hospital, and the number of days of travel reported. Quantification of miles costs performed based on Mileage Allowance Payments established by the UK Government^2^ (45p – rate per business mile for Cars and Vans, first 10,000 miles).

Based on self-reported information, average weekly expenditures were estimated for all carers answering the survey. Moreover, because not all carers are exposed to all costs, average weekly expenditures were also computed for each subset of carers who reported costs greater than zero in each cost category.

Moreover, three heterogeneity analysis were considered for different subsamples. First, we looked at carers with on-site accommodation versus carers who slept at home. Information was available for most participants regarding whether they slept at the hospital (either at bedside or at other hospital-provided facilities), or at home. Information on housing arrangements was missing for only five carers, who were excluded from this specific analysis.

Second, we compared average weekly expenditures for single parent families, and households with both parents. The survey did not include a specific question regarding this. Instead, classification into “single parent” or “both parents” was based on the reported information regarding the number of household members and the number of children in the household. The difference between those two variables was interpreted as a proxy of the number of parents in the household, which should be interpreted with caution. Households where the difference between those two variables was equal to one were classified as “single parent”, while households with a difference equal to two were classified as “both parents”. All remaining households were excluded from this specific analysis (five observations).

Third, we compared carers living in most and least deprived neighbourhoods. Deprivation information was collected from the English Index of Multiple Deprivation, provided by the Ministry of Housing, Communities & Local Government. The most updated version of this index corresponds to the year of 2019, prior to the survey administration. This index is an overall relative measure of deprivation, combining seven different deprivation domains for each Lower Super Output Area code (LSOA). Carers answering the survey reported their post-code information. Post-code data was merged with each LSOA code to identify the index of multiple deprivation associated with each carer’s region. Carers living in districts belonging to the first five deciles of the index were considered to be in the “most deprived” group. Carers living in districts belonging to the last five deciles are assigned to the “least deprived” group. Information on post-codes and on the index of multiple deprivation was missing for only six carers, who were excluded from this specific analysis.

### Estimating productivity losses

Productivity losses were estimated using aggregate wage statistics. Information was collected for the median weekly earnings for full-time employees in the United Kingdom in 2020^3^. These data distinguish the median weekly earnings by gender, and for seven different age groups, based on UK Office for National Statistics(ONS).

For each carer, the estimated weekly wage was adjusteded based on the carer gender and age group, according to ONS data. resulting in an estimated average weekly wage per carer of £441.

Productivity losses were estimated for three sets of carers. The first group included all carers who responded to the survey. The second group included the subset of working carers, either with a full-time or a part-time job. The third group included the subset of working carers with at least one missing workday (in the previous week).

## Results

### Descriptive survey results

In total, 71 surveys were completed. Table 1 describes the survey main descriptive statistics. Mean age of carers was 34 years old, with a mean household size of four members. Apart from the child in the hospital, carers had on average one additional child. In the week preceding the completion in the survey, carers reported missing an average of two days of work because of their child’s hospitalisation. Moreover, they travelled on average four days in that week between home and the hospital, and vice-versa.

**Table 1:** Key descriptive statistics for the survey main variables

Variable	Observations	Mean	Standard Deviation	Minimum	Maximum
Age (years)	71	34.3	7.6	16.0	53.0
Household (number of individuals)	71	4.0	1.6	2.0	9.0
Other children (number)	71	1.3	1.5	0.0	8.0
Distance Home-Hospital (miles)	70	26.1	24.1	0.5	131.0
Off work (days)	65	2.2	2.6	0.0	7.0
Travelling home-hospital (days)*	70	4.3	3.7	0.0	24.0

*In the last week

Table 2 details the characteristics of the carers who answered the survey. 82% of carers were mothers, and 73% households had no more than four members. 34% of carers had only one child who, at the time of survey completion, was hospitalised and 35% of carers had another child besides the one staying at the hospital.

The majority of carers were employed, either in full time (52%) or in part time (11%); 37% were not in paid work. Employment status seemed to reflect the carer’s gender: 41% of mothers reported not being in paid work, compared to 15% of fathers.

Most of carers (54%) reported no missing work days in the week preceding survey completion. However, this estimate included a set of carers who reported no employment status. Considering the subset of carers employed (n = 45, 63%), the proportion reporting zero missing days dropped to 34%.

Eight in ten carers reported travelling between home and the hospital at least once in the past week, and almost one third reported travelling between home and hospital daily in the past week. Still, as described below, some carers were able to stay on-site, which contributed to minimise their travelling expenditures.

**Table 2:** Characterisation of carers (survey responses; N = 71)

	Frequency	Percent	Cumulative
*My relationship to my child who is in Alder Hey is*			
Father	13	18%	18%
Mother	58	82%	100%
*How many people live in your household?*			
2	7	10%	10%
3	25	35%	45%
4	20	28%	73%
5	8	11%	85%
6	4	6%	90%
7	3	4%	94%
8	3	4%	99%
9	1	1%	100%
*Apart from your child in hospital, how many other children do you have?*			
0	24	34%	34%
1	25	35%	69%
2	10	14%	83%
3	5	7%	90%
4	4	6%	96%
5	2	3%	99%
8	1	1%	100%
*Are you currently*			
Employed (full time)	37	52%	52%
Employed (part time)	8	11%	63%
Not in paid work	26	37%	100%
How many days have you had to take off work?*			
0	35	54%	54%
2	3	5%	58%
3	2	3%	62%
4	3	5%	66%
5	15	23%	89%
6	2	3%	92%
7	5	8%	100%
How many days have you had to travel between home and the hospital?*			
0	13	20%	20%
1	5	8%	28%
2	9	14%	42%
3	5	8%	49%
4	4	6%	55%
5	9	14%	69%
7	20	31%	100%

* in the last week; 65 valid observations

### Direct out-of-pocket expenditures

Most carers reported expenditures due to their child’s hospitalisation in the week preceding survey completion. Most carers reported food and drink costs (90%), as well as travelling costs (75%). Almost one third of carers reported parking expenditure (35%). Some carers (17%) reported childcare expenditures or accommodation costs (10%).

Table 3 presents average weekly expenditures reported by all carers in the survey. On average, carers reported weekly expenditures of £164, of which, 38% were due to travelling costs, and food and drink costs each, and accommodation, childcare and parking represented 12%, 7% and 5%, respectively. These expenditures represented 37% of the average weekly wage estimated for these carers.

Because not all carers are exposed to all costs, analysis has been undertaken of the subset of carers who reported costs greater than zero (see Table 3). For instance, a carer living in hospital with their child will not have any accommodation costs or substantial travelling expenditures, although some residential carers may also travel home to see the child’s siblings. Looking to the subset of individuals who report costs greater than zero, average weekly travelling expenditures increase from £62 to £83, while food and drink costs increase from £63 to £70. Parking expenditures also increase from £8 to £23. The largest increases occur in childcare costs which increase from an average of £11 to £66, and accommodation expenditures which increase from £20 to £200.

On-site accommodation can be a way to protect carers from facing high costs. Additionally, on-site accommodation allows carers to be closer to their child, should the child deteriorate, and they need to return to the ward. Information was available for most participants regarding whether they used accommodation at the hospital (either at bedside or at other hospital-provided facilities), or slept at home (see Table 3). Note that not all carers who used hospital accommodation had access to hospital accommodation during the entire length of stay of their child. Most carers (58%) were able to stay in the hospital. The average weekly cost for these carers was substantially lower than the cost faced by carers sleeping at home (£141 versus £212). Carers sleeping at home reported considerably higher travelling, parking, and accommodation expenditures. Conversely, they also reported lower expenditure related to childcare, and food and drinks. Carers without hospital accommodation were financially strained, relative to other carers. In fact, their out-of-pocket expenditures corresponded to 48% of the average weekly wage – above the burden faced by those carers with hospital accommodation (their out-of-pocket expenditures corresponded to 32% of the average weekly wage).

**Table 3:** Out-of-pocket expenses for carers by type of cost (£)

	All carers (N = 71)			Carers reporting costs greater than zero (N = 64^4^)			Carers with hospital accommodation (N = 38)			Carers without hospital accommodation (N = 28)	
Cost category (mean, SD)	Cost	SD		Cost	SD		Cost	SD		Cost	SD
Travelling	62	87		83	90		34	41		103	117
Parking	8	18		23	22		5	10		13	25
Accommodation	20	66		200	99		7	34		42	94
Food and Drink	63	56		70	55		77	65		50	38
Child Care	11	35		66	49		17	45		4	13
Total weekly cost (mean)	164			443			141			212	
% of weekly wage	37%			100%			32%			48%	

Table 4 provides some heterogeneity analysis on the previous results by looking to two particular subsets of carers. On one hand, we decompose the sample between households with a single parent and households with both parents. Households were classified as “single parents” or “both parents” according to the reported information on the number of household members and the number of children. On average, single parents report weekly expenditures of £209 above the expenditures reported by two-parents’ households (£149). Single parents’ expenditures, relative to two-parents’ households, are mostly driven by higher accommodation, travelling and food and drink costs.

On the other hand, the sample was divided between carers living in most and least deprived neighbourhoods. Carers living in districts belonging to the first five deciles of the index of multiple deprivation were considered to be in the “most deprived” group. Carers living in districts belonging to the last five deciles were assigned to the “least deprived” group. According to this classification, carers living in the most deprived regions report 12% lower weekly costs than carers living in the least deprived regions (£162 versus £183). Such decrease is driven by lower travelling and accommodation costs.

**Table 4:** Out-of-pocket expenses for carers according to household and deprivation (£)

	Single parents (N = 19)			Both parents (N = 47)			Most deprived (N = 46)			Least deprived (N = 19)	
Cost category	Cost	SD		Cost	SD		Cost	SD		Cost	SD
Travelling	83	140		57	57		58	98		87	70
Parking	13	28		8	13		12	21		4	10
Accommodation	48	108		17	56		27	72		32	90
Food and Drink	86	71		64	48		68	54		68	63
Child Care	8	16		16	44		11	32		9	25
Total weekly cost (mean)	209	209		149	99		162	135		183	165
% of weekly wage	47%			34%			37%			42%	

### Productivity losses

Productivity losses include work absenteeism for the carers of children who had an in-hospital deterioration. From a societal perspective, there is a significant opportunity cost associated with the days those carers take off work. This opportunity cost was estimated based on average weekly UK wages adjusted by gender and age, since the survey did not include specific questions on wage or income.

Productivity losses do not represent actual financial losses for carers, even though they can be interpreted as a proxy. Losses were computed based on median gross values. This does not consider taxation or social security contributions. To some extent, carers are expected to have some level of protection regarding their absence from work. Still, as gross wages should reflect the marginal productivity of each worker, these estimates should be seen as a proxy for the impact on society of having that worker absent from work.

On average, the weekly productivity loss amounts to £124 per carer. This estimate was performed considering all answers to the survey and corresponds to 28% of the average weekly wage estimated. However, as described above, 37% reported not being in paid work. The remaining carers reported having either full-time or a part-time employment. Focusing on this subset of working carers (n = 45, 63%), the average weekly lost wage increases to £195 (44% of the average weekly wage). Within this subgroup, some carers reported missing work for some days, while others did not report any missing day. In fact, 66% of working carers had missed at least one workday in the previous week. For this subset (n = 29, 41%), the average weekly lost wage increases to £303 (69% of the average weekly wage).

## Discussion

Unplanned critical care admission for children following critical deterioration imposes a substantial burden on carers. The impact of such admission is more than psychological, representing substantial out-of-pocket expenses. Moreover, productivity losses imply a subsequent loss to society. In this study, we provide an assessment on both direct out-of-pocket expenditures and indirect productivity losses. This financial burden is estimated based on 71 carers whose children, following a CDE, were admitted to a critical care unit in a tertiary children’s hospital in the UK. Estimates suggest weekly average direct out-of-pocket expenditures of £164 and a weekly productivity loss for working carers of £195. The magnitude of these estimates is considerable, particularly when considering public policy related with childcare. For instance, the “Universal Credit” policy, established by the UK government to support carers with childcare costs, caps monthly payments to £646 per child, which corresponds to a weekly £162 subsidy. This is closely aligned with the direct out-of-pocket payments estimated for carers of children hospitalised in critical care units.

This paper reinforces previous findings of substantial costs for carers caring for hospitalised children [12, 14–16]. The emergency and unplanned nature of these admissions to critical care means that carers have less time to plan family and friend support. This can in turn accentuate the carer’s financial burden. Results are aligned with the results found by [8] in Switzerland, in which carers insured by the country’s social security system, were found to experience significant non-medical costs during their child’s admission to a paediatric intensive care unit. In our study, undertaken in the UK health system, we provide similar evidence of increasing costs for carers of children admitted to critical care units following in-hospital deterioration. The main sources of expenditure for carers in our study were travel and food and drink, as are seen in [8].

The scope of this study is focused on the assessment of out-of-pocket expenditures and productivity losses experienced during the child’s unexpected hospitalisation in a critical care unit. We do not document any potential long-run costs after the critical care hospitalisation. However, the literature suggests that these losses may continue following critical care hospitalisation. For instance, children often miss school in the weeks following hospital discharge, while carers also miss work [19]. Hence, the estimates of this paper should be interpreted as a lower-bound of the true impact, or as short-run estimates.

Moreover, this paper contributes to the debate on how childhood illness can impose significant costs on carers. As discussed by [20], these costs are not routinely captured by paediatric economic evaluations, which can lead to bias in the analysis.In this study setting the hospital provides subsidised hospital parking and on-site accommodation for some carers with the aim of minimising some direct out-of-pockets expenses. Accommodation is prioritised for carers who live furthest from the hospital or for children who are seriously ill. Nonetheless, the overall impact for carers remains high. Moreover, our estimates should be interpreted as a lower bound when comparing with hospitals without such schemes. These results stress the importance of specific policies to address the financial burden imposed on carers. These policies should be flexible to prevent substantial bureaucratic and time-consuming processes experienced by carers [14].

Results suggest that the relative importance of out-of-pocket payments is different depending on whether carers are able to stay at the hospital or not. Policies aiming at reducing this burden should be adapted depending on the housing arrangements. For carers having to return home, travelling, parking and accommodation expenditures are relatively more important compared with carers staying at the hospital. Nonetheless, even for carers with on-site accommodation, there are periodic costs for returning home to see other family and collect supplies. Interestingly, accommodation expenditures are higher for carers without hospital accommodation, which may reflect that some carers stayed temporarily in accommodations closer to the hospital.

Moreover, results indicate that the financial burden is higher for carers who are single parents, relative to carers that live in two-parent households. Out-of-pocket average weekly expenditures for single parents are 40% higher relative to expenditures reported by two-parent households. This disproportional burden may reflect the struggle faced by single parents when managing simultaneously their hospitalised child with their other arrangements, which may include additional children.

Finally, we found no evidence that carers living in deprived neighbours experience higher out-of-pocket costs than carers living in less deprived regions. In fact, carers living in the most deprived regions report 12% lower weekly costs. This reduction can be explained by different factors. First, we may have different underlying needs and traveling arrangements between both groups. Secondly, specific support provided by charities, social security, and other institutions to deprived carers may be in place, which can help minimising their expenditures. Finally, the lack of disposable income may prevent higher expenditures, crowding-out out-of-pocket expenditures beyond their desired level. However, the assignment of carers to more or least deprived neighbourhoods was made solely based on self-reported post-code information. No information specific to each individual socio-economic condition was collected, which may be different from the average deprivation of the neighbourhood.

This study has some limitations, given the setting and context in which the survey was implemented. First, a retrospective questionnaire was implemented to investigate carers’ expenditures when a child was admitted to the hospital. However, this type of tool is subject to recall errors and selection bias. Secondly, the study is specific in the context of the UK NHS, and a specific hospital. Thirdly, the number of carers included in the study was limited. Additionally, cost information was collected based on self-reported information within cost categories. These do not include other expenditures and are restricted to a specific time frame – the week preceding survey completion. Finally, productivity losses were estimated based on national wage statistics and do not capture specific socio-economic characteristics of the carers or regions where they are located. Since no information regarding carers’ professions was available, productivity losses were estimated adjusting only for age and gender composition.

Moreover, estimates can be affected considering that the study took place during the COVID-19 pandemic, which can introduce some bias in the analysis. In fact, the COVID-19 crisis shifted the usual pattern for carers, given changes in hospital policy for visitors. For instance, visiting periods were restricted during lockdowns, with carer attendance limited to one person per child. Also, hospital parking costs were waived temporarily during the pandemic. Changes to the carers working status during the pandemic, such as wage losses and remote working, may also have affected carers’ out-of-pocket expenditures.

In the UK, the overall number of bed days in paediatric intensive care units was 143,533 in 2019 [21]. Our study estimates a weekly direct out-of-pocket cost per carer of £164, as well as an average productivity loss of £124. These estimates may not be directly comparable with the national context, given the specificities and limitations discussed above. Nonetheless, if one abstracts from those limitations, such financial burden estimate would be translated into a yearly £3.4 million direct out-of-pocket payments paid by carers of children hospitalised in critical care units. Moreover, the national productivity loss associated with those carers would amount to £2.5 million.

Overall, few studies exist regarding the economic burden associated with children’s hospitalisation and even fewer focus on admission to critical care. The present study, despite its limitations, provides evidence that the burden is considerable. Policies and innovations that can reduce the likelihood of critical deterioration events can prevent some of these costs, by reducing or avoiding admissions to critical care units.

Further studies should be developed to inform policy makers on the economic burden faced by carers. These can be crucial to help the design and implementation of policies to support carers during their children’s admission to critical care units.

## Conclusion

This study provides a snapshot of the impact on 71 carers of their child’s stay in a critical care unit in a children’s hospital. Direct out-of-pocket costs for carers are substantial (£164 per week), mostly driven by travelling and food and drink expenditure. Sizeable differences are identified when comparing costs for carers that can stay at the hospital, with carers who had to travel to and from their homes. Moreover, weekly productivity losses for working carers are estimated at £195. The direct out-of-pocket payments, coupled with the indirect productivity losses impose a sizeable economic burden associated with the admission of such children to critical care units.

These results support the development and implementation of policies aiming at reducing the financial burden faced by carers. It also suggests the importance of looking to the economic burden when studying innovations likely to reduce the length of stay in these critical care units.

### Electronic supplementary material

Below is the link to the electronic supplementary material.

Supplementary Material 1

## Data Availability

The datasets used and/or analysed during the current study are available from the corresponding author on reasonable request.

## Footnotes

1 The survey was developed by Céu Mateus, Enitan Carrol, Gerri Sefton, Bernie Carter and carers and approved by the Study Management Group as part of the DETECT project.

2 https://www.gov.uk/expenses-and-benefits-business-travel-mileage/rules-for-tax.

3 https://www.ons.gov.uk/employmentandlabourmarket/peopleinwork/earningsandworkinghours/datasets/agegroupashetable6.

4 The number of carers reporting costs (greater than £0) changes depending on the cost category. The number of observations refers to the category with the largest number of carers reporting costs.

## Declarations

### Disclaimer

This study/project is funded by the NIHR [Dynamic Electronic Tracking and Escalation to reduce Critical Care Transfers (The DETECT Study) (I4I Challenge award ref II-LA-0216-20002)]. The views expressed are those of the author(s) and not necessarily those of the NIHR or the Department of Health and Social Care.

## List of Abbreviations

CDE: Critical Deterioration Event
DETECT: Dynamic Electronic Tracking and Escalation to reduce critical Care Transfers
LSOA: Lower Super Output Area code
NHS: National Health Service
PPI: Patient and Public involvement
