# Supplementary material for: The economic burden experienced by carers of children who had a critical deterioration at a tertiary children’s hospital in the United Kingdom (the DETECT study): an online survey
Source: BMC Pediatr. 2023 Aug 31;23:436. doi: 10.1186/s12887-023-04268-8 (PMC10468882; doi:10.1186/s12887-023-04268-8)
Supplement: Supplementary file 1 — Supplementary Material 1 [file 12887_2023_4268_MOESM1_ESM.docx]

**Appendix 1: Survey flow**

**Q1** I understand that if i tick YES, I am agreeing to take part in this survey. If I don't want to take part I know I can just hand the tablet back to the researcher. *Yes / No*

**Q2** Please enter your study number in the box below. This is the number the nurse/ researcher gave you and it will look like xxxxxxxx.

**Q3** My relationship to my child who is in Alder Hey is: *Mother/Carer / Father/Carer /Other (please explain)*

**Q4** What year were you born in? (This information helps us with our analysis)

**Q5** How many people live in your household?

**Q6** Apart from your child in hospital, how many other children do you have?

**Q7** How far from the hospital do you live (please state in miles)

**Q8** Are you currently: *employed (full time) / employed (part time) / not in paid work / retired*

**Q9** How many days has your child been in hospital during this admission?

**Q10** How many days have you had to take off work?  In the last week (Please tell us to the nearest half day)

**Q11** How many days have you had to come to and from home to the hospital? In the last week.

**Q12** How much money have you spent on travel to and from the hospital per day?

**Q13** If you can't remember how much you've spent, how many miles have you travelled per day (approximately)? In the last week.

**Q14** How much have you spent on parking at the hospital per day? In the last week.

**Q15** If you had to pay for accommodation, how much have you had to pay? In the last week.

**Q16** How much have you spent on food and drink at the hospital per day on average? In the last week.

**Q17** How much more are you spending on child care costs for your other children each day? (If your costs haven't increased or you have no other children just say no extra costs). In the last week.

**Q18** And finally, if you have any other comments about costs you would like to share, please do tell us, we'd love to hear about them.
